# Reconstruction of a large distal femoral giant cell tumor using a 3D-printed condylar support lattice metal implant and fibular grafts: a novel biomechanical and surgical approach

**DOI:** 10.1186/s41205-025-00282-x

**Published:** 2025-07-16

**Authors:** Aashish Chaudhry, Abhishek Kumar Sambharia, Bharat Bahre, Mani Pandey, Tanvi Chawla

**Affiliations:** 1 Department of Orthopedics and Joint Replacement, Aakash Healthcare Superspeciality Hospital, New Delhi, India; 2 Department of Clinical Research, Aakash Healthcare Super speciality Hospital, New Delhi, India; 3 Curewith3D (3D Printing and Designing Company), New Delhi, India

**Keywords:** Distal femoral reconstruction, Giant cell tumor, 3D-printed implant, Fibular grafts, Biomechanical stability, Osseointegration

## Abstract

**Objective:**

This study presents a novel approach to reconstructing a large defect in the load-bearing condylar region of distal femur following the surgical removal of a giant cell tumor (GCT). By using advanced 3D printing technology and virtual surgical planning, designing a patient-specific implant (PSI) to replace the defect and integrate a fibular graft for osseointegration, providing cortical bone strength.

**Methods:**

A 40-year-old female patient with recurrent pain and swelling in the left knee was diagnosed with a distal femoral GCT. Imaging studies confirmed a large lytic lesion with cortical thinning. After tumor excision, reconstruction was performed using a 3D-printed lattice metal implant designed for biological and mechanical integration. A 3D printed custom titanium plate was used additionally for structural support and a fibular graft was embedded within the implant for biological union.

**Results:**

Postoperative outcomes demonstrated progressive osseointegration, weight-bearing capability, and functional recovery. The patient regained maximum osseointegration with the completion of 6 months and full-strength unrestricted mobility at the end of 18 months postoperatively without recurrence. Radiographic follow-ups confirmed structural integrity and graft incorporation.

**Conclusion:**

This study illustrates the successful application of a customized lattice metal implant integrated with a fibular graft, demonstrating its feasibility for large tumor-induced defects in weight-bearing regions.

## Introduction

Bone tumors affecting the distal femur present significant reconstruction challenges due to the region’s load-bearing role and proximity to the knee joint. Conventional reconstruction techniques, including megaprostheses or endoprosthesis and bulk allografts, often present limitations in biomechanical stability and long-term biological integration are often insufficient for large defects [1, 2]. Moreover, achieving adequate functional recovery remains a challenge due to limited osseointegration and potential implant-related complications [2].

Advancements in additive manufacturing or 3D printing have facilitated the development of patient-specific solutions tailored to individual anatomical and mechanical requirements [3, 4]. These techniques allow for the precise replication of bone geometry, offering superior fit and enhanced integration compared to traditional methods. This study reports an innovative approach utilizing a 3D-printed lattice metal implant with embedded fibular grafting and customized titanium plate, aimed at addressing both mechanical strength and biological healing in distal femoral tumor resection cases [5, 6].

## Case presentation

**Fig. 1:**
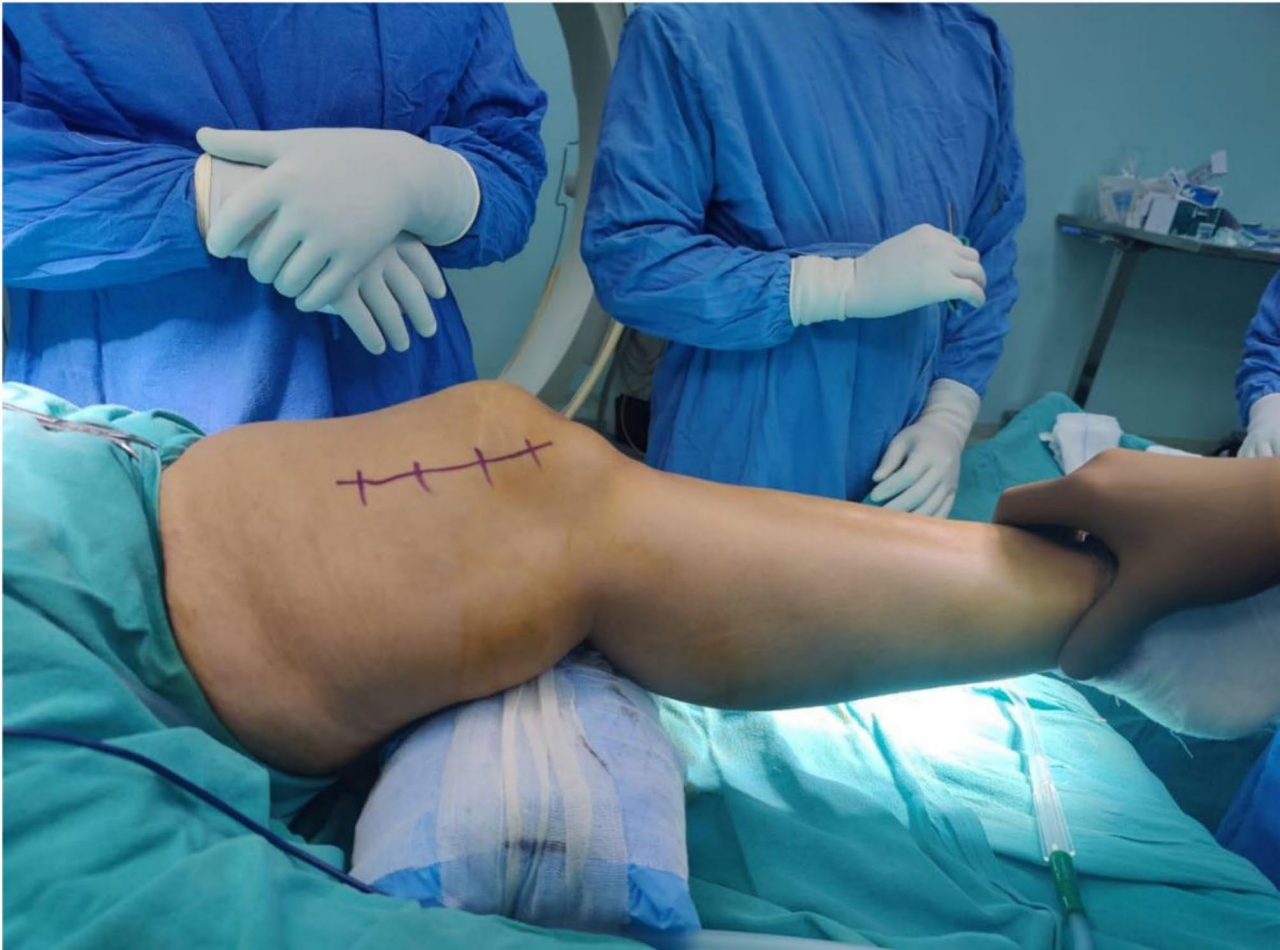
Preoperative image of the patient. A 40-year-old female patient lying on the operating table with the surgical site marked for the procedure. Swelling at the affected area of the left knee is clearly visible, corresponding to the 6-month history of recurrent pain and discomfort. The patient also reported difficulty in weight-bearing and limited ambulation, which led to the decision for surgical intervention

A 40-year-old female presented with a 6-month history of recurrent pain and swelling in the left knee as visible in (Fig. 1), with difficulty in weight-bearing and limited ambulation. The patient had no significant past medical history, and her laboratory investigations were within normal limits, except for mildly elevated inflammatory markers.

### Radiological findings

#### X-ray findings

**Fig. 2:**
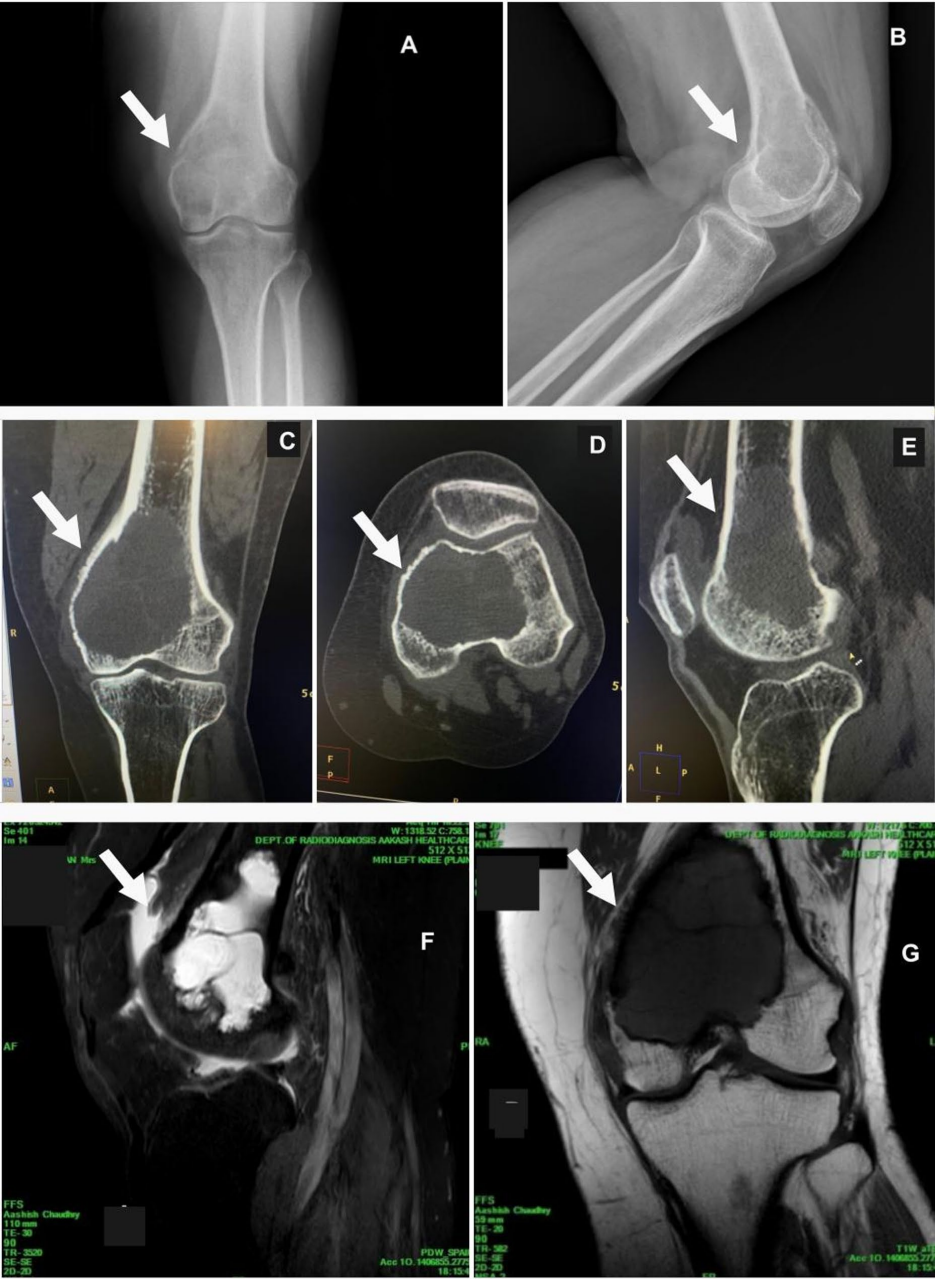
Preoperative radiographic images of the patient with a femoral GCT. **(A, B)**: Show a large, lobulated, lytic, and expansile lesion in the distal femur on X-ray, with a multicystic appearance, internal septations, cortical thinning, and mild periosteal thickening along the medial cortex. The tibiofemoral joint spaces are normal. Differential diagnosis includes giant cell tumor (GCT) or aneurysmal bone cyst (ABC). **(C)**, **(D)** & **(E)**: reveals a similar lesion with multicystic features, posterior cortical destruction, soft tissue bulging, periosteal thickening, cortical thinning, and knee joint effusion. Tibial structures are normal. These findings also suggest GCT or ABC. **(G)** & **(F)**: shows a lesion with fluid-fluid levels, internal septations, and hyperintense signals on T2-weighted/STIR images. Femoral cortex thinning, focal destruction, soft tissue bulging, marrow edema, and mild periosteal thickening are noted, with no signs of metastasis or neurovascular invasion, indicating a benign pathology. The findings are consistent with GCT or ABC, requiring careful surgical planning

Radiographs as in (Fig. 2) showed large, well-defined, lobulated, lytic, and expansile lesion involving the medullary cavity of the distal epiphysis and metaphysis of the left femur. The lesion exhibited a multicystic appearance with internal septations, along with thinning of the overlying cortex and mild periosteal thickening in the metaphyseal region, particularly along the medial cortex. The lesion demonstrated an eccentric growth pattern with minimal surrounding sclerosis, further supporting the suspicion of an aggressive but benign pathology [7, 8]. These features raise the possibility of a giant cell tumor (GCT) or aneurysmal bone cyst (ABC). The lateral and medial tibiofemoral joint spaces on the left side were normal, and no radiographic evidence of a bony loose body was noted. Given the lesion’s characteristics, the differential diagnosis includes GCT or ABC. Clinical correlation is recommended for further evaluation and management [9].

#### MRI findings

MRI of the left knee, performed using a dedicated 16-channel volume knee coil on a 3.0 Tesla scanner, revealed a lesion measuring approximately 66 mm (CC) x 53 mm (TR) x 44 mm (AP) involving the medullary cavity of the distal epiphysis and metaphysis of the left femur. The lesion demonstrated a multicystic appearance with fluid-fluid levels in many of the cystic areas and multiple internal septations. On T1-weighted imaging, the lesion appeared iso to slightly hypointense, while on T2-weighted and STIR images, it showed an intensely hyperintense signal. The lesion caused focal destruction of the overlying posterior cortex of the femur, with a bony defect measuring 15.8 mm x 18.5 mm, and a small soft tissue component measuring 24 mm (CC) x 13.5 mm (AP) x 21 mm (TR), which was bulging posteriorly into adjacent soft tissues with heterogeneous signal intensity [10, 11].

The femoral cortex was thinned, with small areas of full-thickness defects and mild periosteal thickening in the metaphyseal region, more so along the medial cortex. Moderate marrow edema was observed in the distal femur surrounding the lesion, indicative of increased bone activity, while mild to moderate effusion was present in the knee joint, extending into the suprapatellar and retro patellar bursae. Mild diffuse nonspecific soft tissue edema was noted in the intermuscular planes around the distal left femur. The patella, quadriceps tendon, medial and lateral menisci, as well as the anterior and posterior cruciate ligaments, were normal. The tibial condyle and overlying articular cartilage exhibited normal signal intensity.

The findings suggest an aggressive bone lesion, likely consistent with a giant cell tumor (GCT) or aneurysmal bone cyst. There was no evidence of distant metastasis or neurovascular invasion, supporting the feasibility of limb salvage surgery. The lesion’s destruction of the posterior femoral cortex, soft tissue extension, and marrow edema, along with mild knee joint effusion, indicate the need for careful surgical planning and management [12, 13].

#### CT Scan findings

Volumetric scanning of the left knee joint was done using a 128-slice MDCT scanner keeping spital resolution high, slice thickness/increment 0.8, tube voltage 120 kvp, tube current mA 150, gantry rotation time 0.4 s and exposure time of 6.9 s. The CT scan images revealed a large, well-defined, lobulated, lytic, and expansile lesion involving the medullary cavity of the distal epiphysis and metaphysis of the left femur, measuring approximately 66 mm (CC) x 53 mm (TR) x 44 mm (AP). The lesion demonstrated a multicystic appearance with internal septations, with no evidence of calcification or fat attenuation. It caused focal destruction of the overlying posterior cortex of the femur, with a bony defect measuring 15.8 mm x 18.5 mm. Additionally, a small soft tissue component, measuring 24 mm (CC) x 13.5 mm (AP) x 21 mm (TR), was noted bulging posteriorly into the adjacent soft tissues. The overlying femoral cortex showed thinning with small areas of full-thickness defects, and mild periosteal thickening was observed, particularly along the medial cortex of the metaphyseal region. Importantly, the lesion did not extend to the articular surface of the bilateral femoral condyles.

Mild to moderate effusion was noted in the knee joint, extending into the suprapatellar and retro patellar bursae, while mild diffuse atrophy was observed in the visualized muscles of the distal left thigh. CT confirmed the presence and extent of bony cortical destruction and thinning with soft tissue component posteriorly bulging into adjacent tissues supporting the diagnosis and area involvement of a giant cell tumor (GCT) [14]. The remaining bony structures of the knee joint, including the tibial condyle, appeared normal, and the patellofemoral and tibiofemoral joint spaces were intact. These findings are consistent with a differential diagnosis of a giant cell tumor or aneurysmal bone cyst, emphasizing the need for further clinical and radiologic evaluation to guide appropriate treatment, particularly considering the risk of structural instability due to the cortical breaches and soft tissue extension observed. Further, no intra-articular involvement was noted. Cortical breach was evident in multiple areas, raising concerns about structural stability and reinforcing the need for a robust reconstructive strategy [15].

### Surgical approach

A diagnosis of GCT of the left distal femur was established [16]. The patient was planned for extended curettage and reconstruction using a 3D-printed implant integrated with fibular grafting [17].

#### Pre-surgical planning

**Fig. 3:**
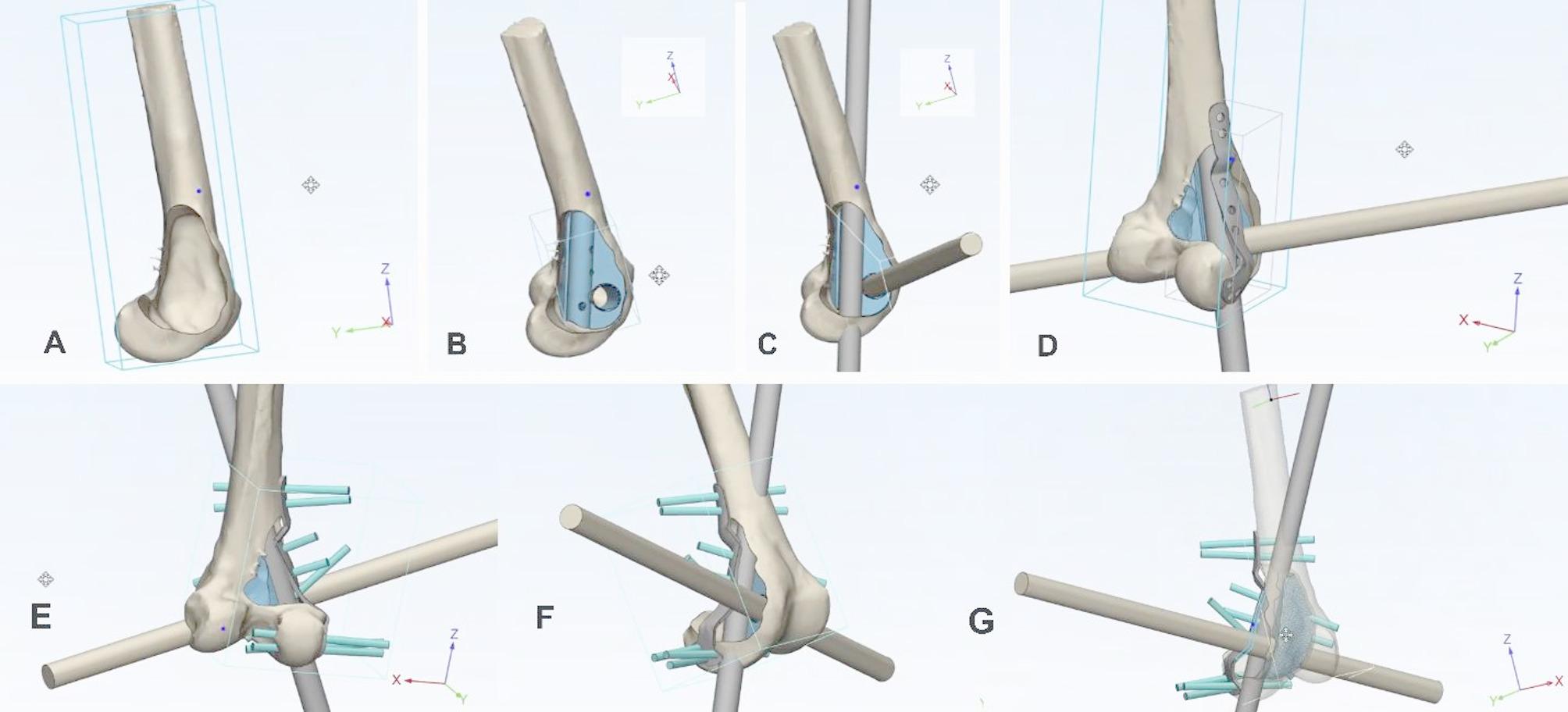
3D virtual pre-surgical planning. **(A)**: showing the distal femoral area after the removal of a giant cell tumor, with the cavity clearly visible in the 3D design created virtually from the radiograph design. **(B)**: It displays the placement of a lattice metal implant inside the cavity, designed to match the cavity space. **(C)**: shows two rod-like structures, representing a fibula graft embedded inside the lattice metal implant to facilitate osseointegration. **(D)**: 3D illustration of a metal plate positioned on the lattice metal implant and fibula, to be secured with screws. **(E)**: 3D representation of the placement of screws to secure and provide strength to the implant and fibula. **(F)**: Complete 3D view showing the metal plate, lattice metal implant, and fibula graft with screws tightened, providing enhanced stability for better osseointegration. **(G)**: Full mechanical and biological virtual structure with transparency to aid in understanding the placement and design of the implant

**Fig. 4:**
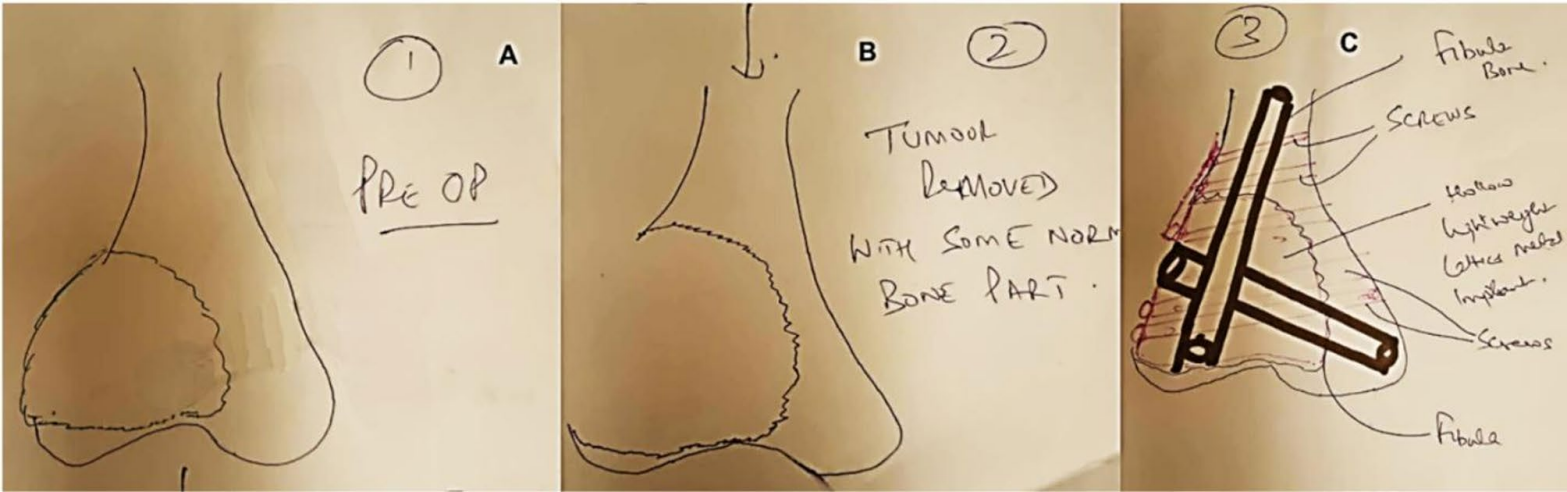
Handout planning used before virtual design planning. **(A)** Preoperative image showing a giant cell tumor at the distal femur. The tumor’s location and extent are clearly visible, highlighting the area requiring surgical intervention. **(B)** Postoperative image following the removal of the giant cell tumor, with the resulting cavity visible. The tumor has been excised, and the empty space is evident in the femoral region. **(C)** Virtual planning on paper for the surgical reconstruction, showing the placement of screws, implant, and tibial graft. This plan outlines the optimal positioning for stabilizing the area after tumor removal

**Fig. 5:**
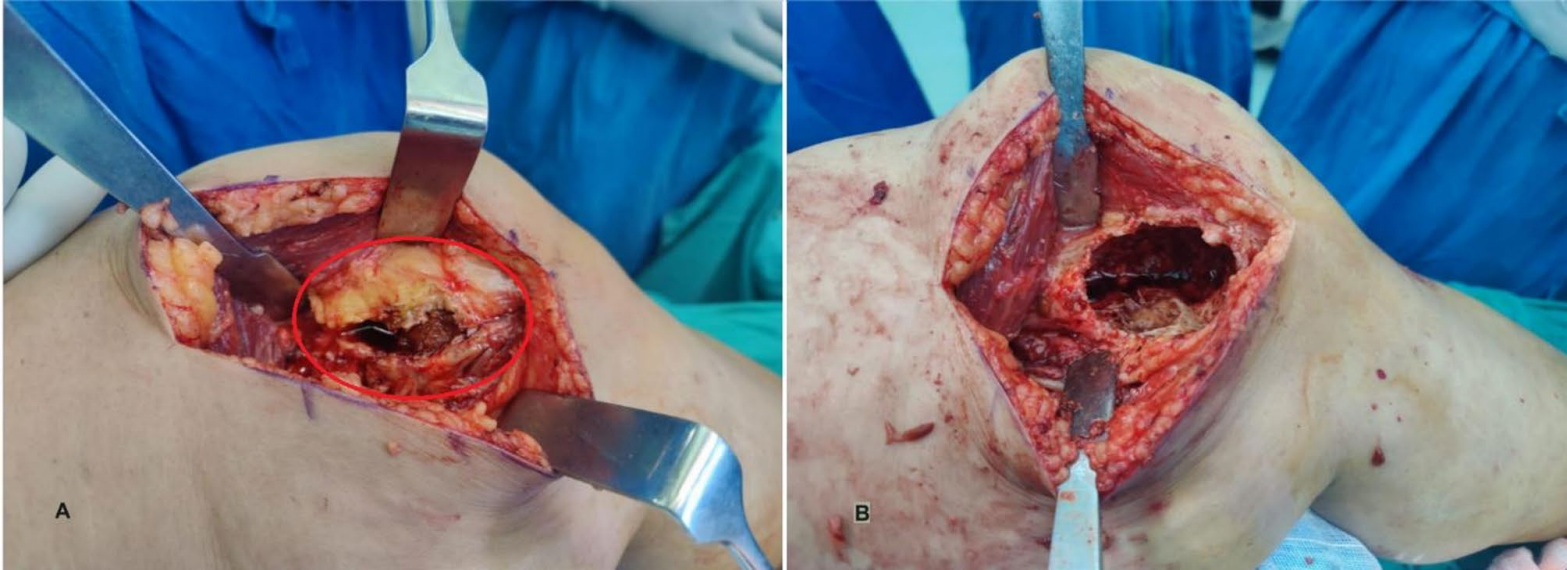
Intraoperative images before and after tumor removal. **(A)** Intraoperative image showing the exposed giant cell tumor following a medial approach excision. **(B)** Intraoperative image showing the smoothened cavity after tumor excision for implant and fibular graft insertion to create a hybrid mechanical-biological construct

1. **Virtual surgical planning**: Based on radiological findings a patient specific approach was planned as depicted in (Figs. 3 and 4). A 3D model was planned with the aid of 3D design software ‘Materialise 3-matic Medical’ designed using CT and MRI imaging dicom data to assess tumor extent and design the implant [18, 19]. Mechanical and chemical characterization of the Ti6Al4V alloy implant, confirmed by both manufacturer through third-party laboratory reports (reports available on request), demonstrated compliance with American Society for Testing and Materials (ASTM) Grade 23 and F136 standards. The implant exhibited a tensile strength of up to 1185.7 N/mm², yield stress of 1179.2 N/mm², elongation of 10.96%, and a modulus of elasticity of 114 GPa, indicating its suitability for withstanding physiological biomechanical loads [20].

2. **Customized implant design**: A lightweight lattice structured titanium metal implant made of rigid titanium alloy Ti-6Al-4 V (Titanium alloyed with 6% Aluminum and 4% Vanadium) using Direct Metal Laser Sintering (DMLS) technology with an EOS M290 printer as in (Fig. 8) was designed to fit within the resected cavity (Fig. 5) accommodating an embedded fibular graft to promote osseointegration [21, 22].
3. **Mechanical and biological construct**: The lattice structure was not solely intended for weight bearing but was designed to promote biological fixation [12, 23]. The fibula graft (20 cm) was harvested from the ipsilateral side, divided into two, and embedded into the implant with titanium plate alongside artificial bone grafting (Genex) to fill residual defects for enhanced biological integration. This combination showed in (Figs. 3 and 4) aimed to balance mechanical load distribution while stimulating natural bone regeneration [24].

### Surgical procedure

#### Anesthesia & positioning

The patient is positioned in a supine (lying on their back) position on the operating table. Regional Anesthesia is induced and maintained. A pneumatic tourniquet is applied to the upper thigh of the left leg flexed at 30° to control blood flow and minimize blood loss during the procedure with pressure properly monitored for safety and effective hemostasis [25]. Careful patient positioning ensured optimal surgical access while minimizing pressure points and nerve compression risks [26].

#### Surgical steps

The tumor as visible in (Fig. 9) was excised via a Medial approach; an incision of 10 cm was made along the medial joint line. Excision of tumor was done with lesion was curetted and debrided, and the cavity as visible in (Fig. 9) was smoothened using a burr. Chemical cauterization with absolute alcohol was applied to the cavity to reduce recurrence risk [27, 28]. The patient specific customized 3D-printed lattice metal implant was inserted into the cavity. A fibular graft, divided into two sections was inserted into the implant slots to create a hybrid mechanical biological construct. For final fixation a customized locking titanium plate was used for structural support. Then closure & immobilization of wound done in layers, and an above-knee slab was applied [20].

### Post-operative care

Post-operative care began after the successful completion of the surgery and suturing, as shown in (Fig. 9), which presents the Post-Op Day 1 radiograph image of the surgical site.

**Fig. 6:**
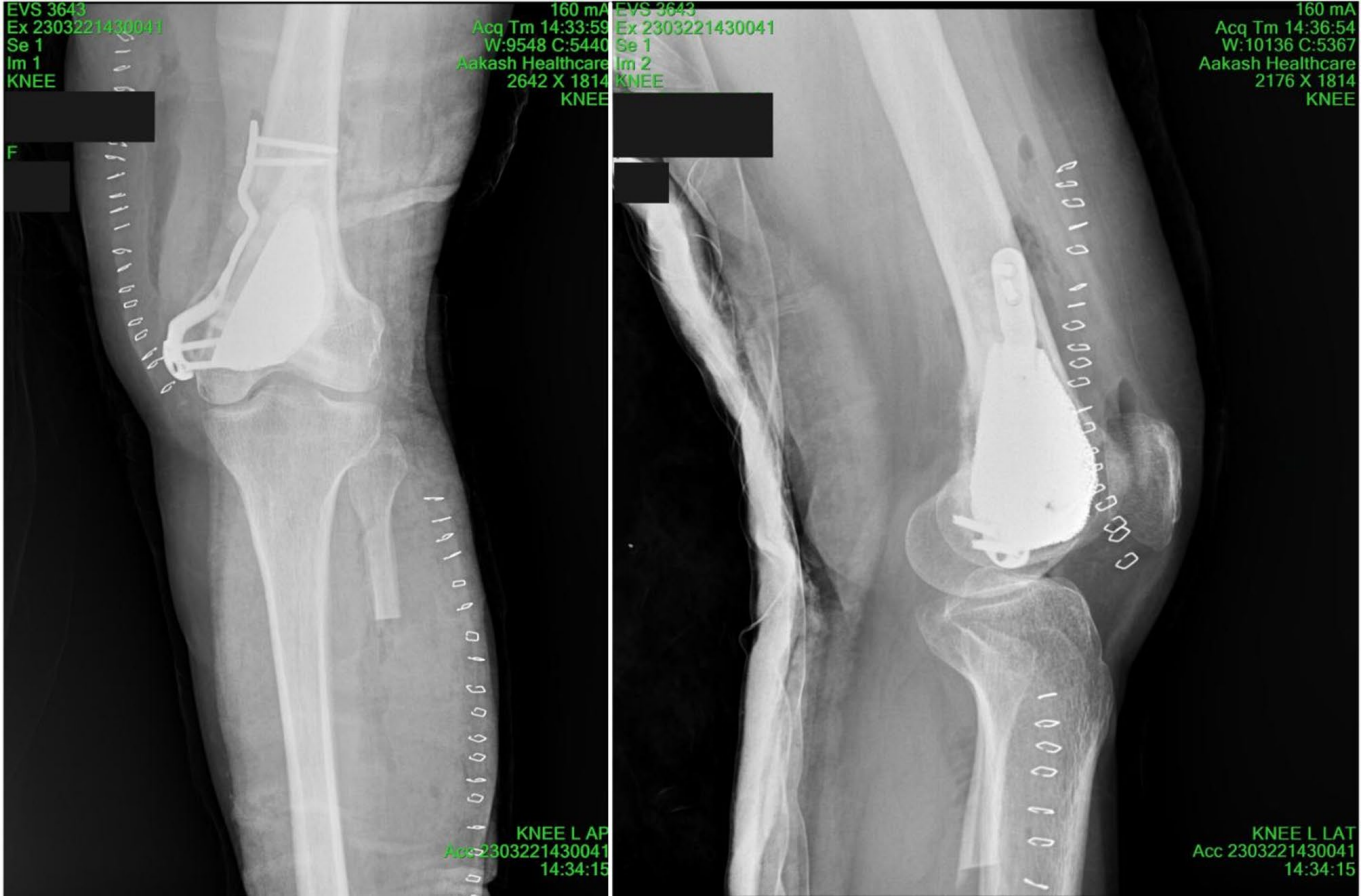
Post-operative radiograph of the left femur. X-ray image taken post-op immediately after surgery, showing the surgical site, after suturing and bandaging, displaying the surgical site post-procedure

**Fig. 7:**
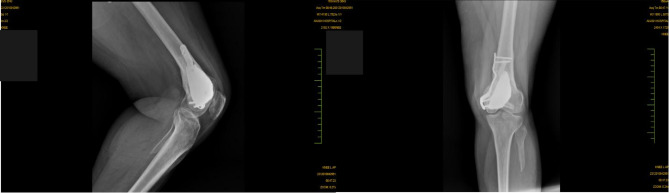
Radiographs taken at 6 months post-surgery. It shows complete structural integration of the affected area. The images confirm that the healing process is fully completed with no recurrence of complications. This stage marks the successful restoration of the patient’s mobility

The patient was administered intravenous fluids, antibiotics, and analgesics to manage hydration, prevent infection, and control pain, respectively. Supportive measures were also provided to ensure a smooth recovery. Deep vein thrombosis prophylaxis was administered using low-molecular-weight heparin to mitigate thromboembolic risks. By Day 17, the wound healing was noted to be satisfactory, with no signs of complications. On Day 23, progressive mobilization was initiated, allowing for gradual movement and rehabilitation to restore function [29]. Physical therapy was focused on quadriceps strengthening and knee range-of-motion exercises to prevent joint stiffness. At 6 weeks, the patient was able to bear full weight on the affected leg with the aid of a walker. By 4 months, the patient transitioned to using a stick for walking, and a knee hinge brace was introduced to provide additional support and stability [15]. Post completion of 6 months, the radiographs as in (Fig. 7) confirmed that the structural integration of the affected area was complete and without recurrence of any issues during the follow-up period. Upon completion of 18 months, the patient was cleared for full activity [30].

## Materials and methods

### Innovative design features

#### Lattice metal implant

**Fig. 8:**
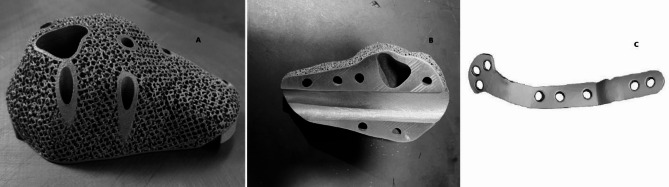
Implants used in the surgery. **(A)** Lattice metal implant porous design with pore size osity of 1.0–1.5 mm showcasing its lightweight yet mechanically stable structure. The implant’s unique porous architecture allows for osseointegration, promoting bone tissue growth into the implant, thereby enhancing the bond between the implant and surrounding bone. This design is ideal for applications requiring both strength and lightweight properties while ensuring long-term stability and function. **(B)** Detailed view of the lattice metal implant’s internal structure. The design facilitates the integration of bone tissue, which contributes to the implant’s stability and durability. This architecture provides an optimal balance of mechanical support and biological integration, ensuring long-term success in orthopedic applications. **(C)** Custom-designed titanium fixation plate stabilizing the injured or reconstructed area during healing. Known for its biocompatibility, it ensures secure alignment, supports bone healing, and minimizes adverse reactions. Its durability withstands mechanical stresses, maintaining proper alignment and enhancing long-term surgical success

The lattice metal implant is designed as in (Fig. 8) to reduce weight while maintaining mechanical stability, making it an ideal choice for applications where both strength and lightweight properties are crucial. Its unique porous architecture with porosity of 1.0–1.5 mm facilitates osseointegration, allowing bone tissue to grow into the implant, promoting a strong and lasting bond between the implant and the surrounding bone. This structure not only supports the mechanical needs of the body but also enhances the biological integration, providing long-term stability and function [31]. The use of titanium alloy ensures corrosion resistance and durability, critical for long-term implant success [32].

**Fig. 9:**
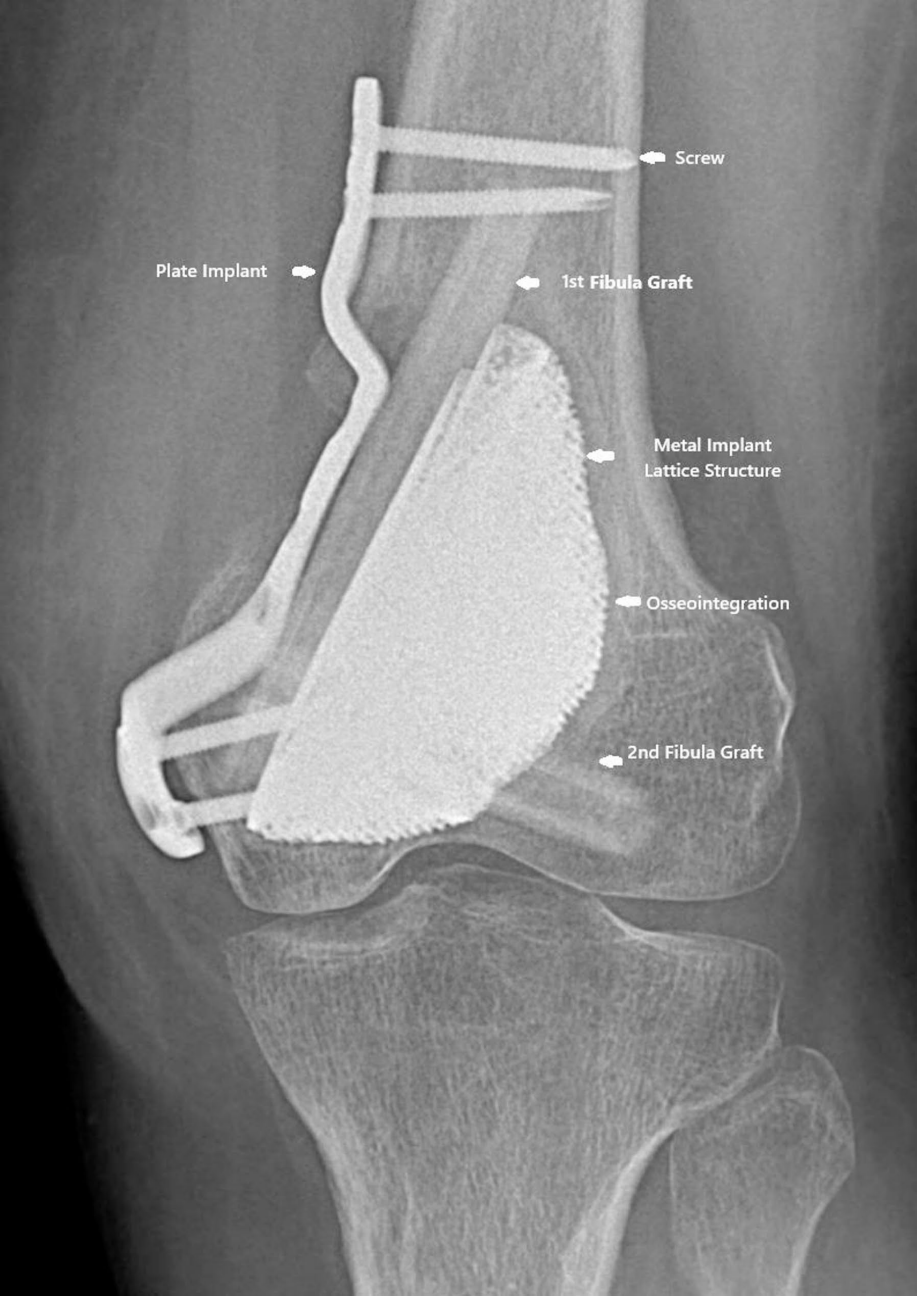
Femoral area visualization after 6 months of osteointegration

Fibular integration plays a crucial role in providing cortical bone strength, ensuring the structural integrity of the affected area. By facilitating enhanced osseointegration, it promotes the bonding of the implant with the surrounding bone, which contributes to improved stability and long-term function. This integration as visualized in (Fig. 9) also supports efficient load transfer, allowing forces to be evenly distributed across the skeletal structure, which is essential for maintaining balance and minimizing stress on the healing tissues [33]. The result is a more robust and functional connection between the implant and the bone, aiding in the patient’s overall recovery and mobility [24, 29].

The titanium fixation plate, porous metal implant, and fibular integration with screws could be seen forming hybrid mechanical-biological construct post six months. This design enhances cortical bone strength, promoting osseointegration for a strong bond between the implant and surrounding bone. Efficient load transfer ensures even force distribution, maintaining balance and reducing stress on healing tissues, resulting in a stable, functional connection that supports recovery and improved mobility.

### Titanium fixation plate

In the design, the titanium fixation plate component as visualized in (Fig. 9) ensures the stability of the injured or reconstructed area, providing secured alignment and support for the bones during the healing process. Made from titanium, it offers excellent biocompatibility, which minimizes the risk of adverse reactions and promotes a smooth recovery [31]. The plate is designed to support long-term mechanical integrity, withstanding the stresses and forces placed on the bone as it heals and regains strength. Its durability and strength help maintain proper alignment over time, contributing to the overall success of the surgical intervention and enhancing the long-term functionality of the affected area.

#### Ethics statement

This surgical case, involving the treatment of a giant cell tumor in the left radial femoral part using a 3D-printed implant and fibular graft for osseointegration, was performed as part of a personalized treatment plan tailored to the patient’s condition. Due to the lack of suitable alternative conventional methods that could support better patient outcomes, the use of a 3D-printed customized implant was considered an innovative and necessary solution. Although Institutional Review Board (IRB) approval was not sought at the time of surgery due to the unique, personalized nature of the treatment and the urgent clinical need, the patient provided informed consent for the use of the 3D-printed customized implant and for the publication of their case as an original article. All patient identifiers were removed to maintain confidentiality. his study was conducted in accordance with the ethical standards of Aakash Healthcare Superspeciality Hospital, a quality-accredited institution with NABH (National Accreditation Board for Hospitals & Healthcare Providers) certification, and the Declaration of Helsinki. All clinical protocols were approved by the institutional clinical committee. The research adhered to the guidelines of the International Committee of Medical Journal Editors (ICMJE), maintaining principles of transparency, integrity, and scientific rigor. Informed consent was obtained from the patient, and all procedures were carried out in compliance with relevant guidelines and regulations. The case is now being shared to contribute knowledge to the field and offer an alternative approach for similar clinical scenarios, with successful recovery and excellent treatment outcomes.

## Comparative analysis

The comparative analysis of different reconstruction methods as presented in (Table 1) highlights their varying strengths in terms of mechanical stability, osseointegration, and load-bearing potential.

**Table 1:** Evaluation of stability, osseointegration, and Load-Bearing potential in different reconstruction approaches

Reconstruction Method	Mechanical Stability	Osseointegration	Load-Bearing Potential
Megaprosthesis	High	None	Limited
Bulk Allograft	Moderate	Variable	Limited
3D-Printed Implant + Fibula Graft	High	Strong	Excellent

- Megaprosthesis offers high mechanical stability but lacks osseointegration potential, meaning it does not biologically bond with the bone. Its load-bearing potential is limited, making it suitable for situations requiring stability but less effective in long-term, functional load distribution [13, 34].
- Bulk Allograft provides moderate mechanical stability and variable osseointegration depending on the graft’s quality and integration with the patient’s bone. Its load-bearing potential is limited, as the graft may not consistently handle stresses over time, especially in weight-bearing applications [34–36].
- 3D-Printed Implant + Fibula Graft combines high mechanical stability with strong osseointegration, offering a biologically integrated solution that supports long-term healing. Its load-bearing potential is excellent, making it ideal for supporting the affected area through both early recovery and full functional use [26, 32, 37].

## Result & discussion

The wound healing process showed no clinical signs of infection or other complications. Progressive mobilization commenced on Day 23, facilitating gradual restoration of limb function. Physical therapy focused on strengthening the quadriceps and maintaining knee range of motion to prevent postoperative joint stiffness. At the 6-week mark, the patient achieved full weight-bearing capability on the affected limb with the assistance of a walker. By 4 months, the patient transitioned to ambulation with a walking stick, and a knee hinge brace was introduced to enhance joint stability and support. Follow-up radiographs at 6 months confirmed complete structural integration of the treated area, with no evidence of recurrence or delayed healing. At the 18-month follow-up, the patient demonstrated full recovery and was cleared for unrestricted activity.

## Conclusion

The integration of virtual planning, 3D printing, and biological augmentation offers a promising approach for complex femoral reconstructions. Above customized approach of a 3D-printed lattice metal implant with an embedded fibular graft offers a promising solution for large distal femoral defects post-tumor excision. Radiographs taken at 6 months post-surgery showed complete structural integration of the affected area. The combination of biomechanical support and osseointegration resulted in a successful clinical outcome with full weight-bearing restoration and no recurrence at 18 months [30, 33]. The application of above surgical approach highlights its significant potential as a viable alternative to conventional reconstruction methods in orthopedic oncology and personalized reconstruction [38, 39].

In summary, the 3D-printed implant with fibula graft provides the best overall performance in terms of mechanical stability, osseointegration, and load-bearing potential [40]. This novel approach demonstrates the advantages of combining customized additive manufacturing with biological augmentation for enhanced functional recovery [38].

## Data Availability

No datasets were generated or analysed during the current study.

## Abbreviations

GCT: Giant Cell Tumor
PSI: Patient-Specific Implant
3D: Three-Dimensional
ABC: Aneurysmal Bone Cyst
MRI: Magnetic Resonance Imaging
CT: Computed Tomography
IV: Intravenous
LMWH: Low-Molecular-Weight Heparin
CC: Cranio-Caudal (measurement plane)
TR: Transverse-Radial (measurement plane)
AP: Antero-Posterior (measurement plane)
STIR: Short Tau Inversion Recovery (MRI sequence)
MDCT: Multidetector Computed Tomography
T1WI: T1 Weighted Imaging
T2WI: T2 Weighted Imaging
NABH: National Accreditation Board for Hospitals & Healthcare Providers
ICMJE: International Committee of Medical Journal Editors
